# Artificial Intelligence (AI) in Pharmaceutical Formulation and Dosage Calculations

**DOI:** 10.3390/pharmaceutics17111440

**Published:** 2025-11-07

**Authors:** Sameer Joshi, Sandeep Sheth

**Affiliations:** 1 Department of Pharmaceutical Sciences, School of Pharmacy, Philadelphia College of Osteopathic Medicine, Suwanee, GA 30024, USA; sameerjo@pcom.edu; 2 Department of Pharmaceutical Sciences, College of Pharmacy, Larkin University, Miami, FL 33169, USA

**Keywords:** artificial intelligence, pharmaceutical calculations, drug formulation, precision dosing, machine learning in pharmaceutics

## Abstract

Artificial intelligence (AI) is reforming pharmaceutical sciences by renovating traditional drug formulation and dosage calculation approaches. This review provides a comprehensive overview of how AI technologies, such as machine learning (ML), deep learning (DL), and natural language processing (NLP), are currently being used in pharmaceutical calculations to improve accuracy, efficiency, and personalization. We have explored the role of AI in predicting drug properties, excipient optimization, and formulation design, as well as its applications in pharmacokinetic/pharmacodynamic (PK/PD) modeling, real-time dose adjustment, and precision medicine. Despite significant progress, data quality, interpretability, regulatory acceptance, and ethical considerations persist. Therefore, this review examines the impact of AI on automated decision-making, quality control, and regulatory compliance in pharmaceutical formulation development. The article also highlights the emerging trends in pharmaceuticals, including AI-assisted 3D printing, integration with wearable technologies, and emphasizing AI’s transformative potential in reforming the landscape of pharmaceuticals and personalized therapeutics.

## 1. Introduction

Calculations are integral to formulate, develop, and administer safe and effective medications. Conventional mathematical models and manual computations are progressively being enhanced by AI, due to its capacity to process vast datasets, identify complex patterns, and assist in decision-making. AI can transform pharmaceutical calculations from conventional, static models to dynamic, adaptive systems. The significance of this transformation is in the precision, clarity, and efficiency that AI offers, especially in areas of variance and complexity; for example, personalized dosing and formulation optimization are critical. This review explores several facets of the application of AI in calculations, focusing on formulation development and dosage determination.

In the last decade, many industries, including the pharmaceutical industry, have witnessed a surge in the use of data-driven tools. In the pharmaceutical industry, these tools are often fueled by the exponential growth in biomedical data and computational modeling. The ability of AI to handle these high-dimensional datasets, simulate the behavior of molecules, and present the possible therapeutic outcomes has made it an invaluable asset in drug discovery and other downstream processes like formulation development and dosing [1,2,3]. Once purely arithmetic and standard protocol-driven, pharmaceutical calculations transform into intelligent systems capable of delivering patient-specific and compound-specific needs [4]. Additionally, enhancing the scientific rigor of formulation development and dosing determination, these innovative systems also reduce time, effort, and cost in the formulation development process. For example, AI-driven models can quickly optimize excipient combinations and predict dose adjustments in patients with renal impairment, tasks that previously required extensive experimental or clinical trials [2,5]. Efficiencies like this will accelerate therapeutic development and contribute to the broader impact of precision medicine, where treatments are tailored to individual patients based on physiological, genetic, and lifestyle factors [6]. Table 1 summarizes different AI techniques that have potential in pharmaceutical applications. However, this review discusses major AI techniques (e.g., ANNs, SVMs, etc.) applied in pharmaceutical formulation and dosage calculations. Each technique offers unique advantages in prediction, optimization, and decision-making.

Briefly, this review provides an integrated perspective on how AI is revolutionizing pharmaceutical formulation and dosage calculations by bridging computational modeling with experimental pharmaceutics. This review presents a novel AI-based formulation-to-dose continuum, highlights cutting-edge ML techniques applied in dosage design, and addresses regulatory and ethical frameworks essential for clinical translation. While several AI models have demonstrated strong predictive power in preclinical and formulation optimization studies, most applications remain at the proof-of-concept or early translational stage, with limited clinical validation to date. The continued integration of AI with clinical data pipelines is therefore crucial for achieving fully validated, patient-specific dosing strategies. Overall, these insights aim to guide educators, researchers, and industry professionals toward a more informed, data-driven, and patient-specific approach to the development of drugs and their associated products.

## 2. AI in Pharmaceutical Sciences

AIs are computational systems capable of performing tasks that typically require human intelligence, including learning, reasoning, and problem-solving. ML, DL, and NLP are the key AI systems in pharmaceutical sciences. ML algorithms, such as SVMs, decision trees, and neural networks, can identify nonlinear relationships in pharmacological data. Deep learning, a subset of ML, uses multilayered neural networks to model complex patterns in large datasets. This task is instrumental in drug discovery and activity predictions [43,44]. AI contributes beyond computation; it empowers predictive analytics, real-time data monitoring, formulation optimization, and dose determination, offering a level of compliance not previously achievable through conventional means [2,45].

### 2.1. AI in Pharmaceutics

Different branches of AI are used based on the task at hand. Supervised learning is commonly employed for predictive modeling using labeled datasets, whereas unsupervised learning is applied for clustering similar molecules or formulations without predefined labels. Reinforcement learning, though less common, is gaining importance for optimizing sequential decision-making processes such as adaptive dosing [44]. These novel systems simulate human decision-making using rule-based logic and are often integrated into clinical decisions as support tools. In pharmaceutics, NLP is increasingly used to extract relevant information from unstructured data sources like electronic health records (EHRs), scientific literature, and clinical trial reports. This facilitates identifying drug interactions, dosing patterns, and patient-specific factors influencing pharmaceutical calculations [44,46].

### 2.2. Data Requirements and Preprocessing in AI Models

The effectiveness of AI models in pharmaceutical sciences hinges on data availability and quality. High-quality, processed datasets are essential for compatible training models across diverse populations and drug formulations. Each data preprocessing step, such as normalization, feature selection, dimensionality reduction, and outlier removal, is crucial to ensuring the models are robust, rugged, and interpretable [47]. Furthermore, integrating diverse data types ranging from molecular descriptors and in vitro test results to real-world clinical data reflects the model’s ability to record complex pharmacological behaviors. On the other side, the use of federated learning and privacy-preserving techniques is also expanding, allowing researchers to utilize sensitive patient data without compromising confidentiality [48].

## 3. AI in Formulation Development

Over the past 10 years, AI has laid the foundation stone in modern formulation development, enabling optimizations driven by data throughout the development process. Conventionally, formulators/formulation scientists/chemists trusted trial-and-error methodologies following the DoE, which were time-consuming and resource-demanding. With modern ML and predictive intelligence, researchers/compounders can now screen a large number of potential excipients and active pharmaceutical ingredient (API) combinations, predict their compatibility, and finally optimize the formulation parameters, before conducting any laboratory-based experiment [2,4].

Some machine learning models, such as random forest regressors, SVMs, and deep neural networks, have been reported to predict essential and critical formulation parameters like solubility, dissolution rate, hygroscopicity, and polymorphism. Most of these predictions were based on molecular and historical formulation data, which allowed rapid prototyping and a significant reduction in production cost [1].

AI has also improved the selection and optimization of excipients using empirical screening. Bayesian optimization (BO), that balances exploitation and exploration, and generative design algorithms that create multiple design options, are increasingly used to identify ideal combinations of pharmaceutical excipients that enhance drug absorption, bioavailability, and stability while minimizing toxicity or adverse interactions [2]. Moreover, reinforcement learning algorithms are being explored for optimal decisions to dynamically update developmental strategies in response to real-time data, further supporting the concept of adaptive formulation design [49].

Another hopeful application is in silico-DoE, where AI-driven tools help reduce the need for high-throughput experimental runs by generating virtual screening environments. These systems can simulate experimental outcomes under various formulation settings, guiding researchers to focus only on the promising combinations for in vitro or in vivo validation [50]. Using AI in formulation development workflows improves speed, precision, and accuracy, aligning with the regulatory principles such as quality by design (QbD) [51].

AI also helps in the determination of the encapsulation efficiency and the drug release kinetics in advanced formulation platforms such as liposomes, lipid nanoparticles, solid colloidal dispersions, and other controlled-release drug delivery systems. This helps formulation scientists fine-tune the target site, release rate, and degradation profiles. Recently, Li et al. (2020) applied supervised machine learning to liposomal formulations and found improved accuracy in predicting size, polydispersity index (PDI), and encapsulation outcomes in comparison with the conventional models [52].

The future of AI in drug formulation lies in integrating it with automation, robotics, and laboratory information management systems (LIMS) to create autonomous formulation labs or “self-driving laboratories.” These facilities use AI to regularly design, execute, and learn from experiments, thus continuously improvising the formulation strategy with minimal human intervention and errors [1]. The key applications of AI in pharmaceutical formulation development are discussed in further subsections.

### 3.1. Predictive Modeling of Drug Properties

Prognostic modeling of drugs and their properties may be very impactful on pharmaceutical formulation [1,53]. By analyzing large datasets, AI models can provide information on key physicochemical and biopharmaceutical characteristics such as solubility, rate of dissolution, permeability, and chemical stability at various temperatures and humidity. These datasets are not limited to molecular descriptors, but also formulation parameters and biological responses. Modeling of these properties plays a vital role in determining bioavailability, absorption kinetics, and therapeutic efficacy [54,55].

SVMs, RF classifiers, and ANNs are frequently applied to establish nonlinear relationships between chemical structures and their corresponding behaviors in various dosage forms. In recent years, these models have outperformed traditional quantitative structure–activity relationship (QSAR) approaches in accuracy and user compliance. For example, DL models trained on curated compound libraries have demonstrated high predictive power for solubility in multiple solvent systems, enabling balanced selection of drug candidates for further development [1,45,56].

AI also facilitates early screening for formulation risk and poor bioavailability, reducing the likelihood of failure in later stages of product development. Generative models and unsupervised clustering algorithms are now being used to identify molecular scaffolds that meet pharmacodynamic (PD) requirements and show promising formulation characteristics [57]. For example, as demonstrated by Macarron et al. (2011), high-throughput, AI-enabled screening not only accelerates lead compound identification but also informs preformulating studies by predicting solubility, stability, and excipient compatibility, which are critical determinants in developing optimized dosage forms [50]. Moreover, the use of hybrid models that consider other principles of cheminformatics, thermodynamics, and biological data is under development to predict the interaction of formulation components under various physiological conditions [49,58].

The ability of predictive modeling can be amplified when it is coupled with high-throughput experimentation (HTE) or robotics. These AI-enabled systems can alone generate, test, and refine pharmaceutical formulations, simultaneously updating their forecasts based on recent experimental outcomes. This lays the foundation for adaptive formulation platforms and self-learning labs [49,50].

### 3.2. Excipient Selection and Optimization

Excipient selection is a critical step in drug formulation development, which decides the stability, solubility, manufacturability, bioavailability, and patient acceptability. Traditionally, excipient choice depended on experiential screening, prior knowledge, and guidance from pharmacopeia. This approach appeared time-consuming, expensive, and often not capable of capturing complex and nonlinear interactions between active pharmaceutical ingredients (APIs) and other excipients. AI now provides a systematic and solution-rich approach with data to this challenge.

AI systems can suggest suitable excipient combinations that enhance drug performance and stability using historical formulation datasets combined with cheminformatics and material property databases. For instance, supervised learning models (random forest, gradient boosting, deep neural networks) can classify excipients based on predicted compatibility with APIs. In contrast, models based on regression can predict ideal excipient ratios to achieve target dissolution, compressibility, or disintegration times [4,51,59].

Moreover, iterative optimization techniques such as BO and RL are increasingly applied in fine-tuning of the excipient concentrations. These models continuously refine outcome predictions based on recent outcomes, making the predictions more robust compared to traditional DoE approaches. DoE requires pre-specified models and assumptions but AI methods adapt dynamically, capturing nonlinearities and higher-order interactions between excipients and APIs [13].

A notable application is in stability prediction. Neural network-based models have been used to predict excipient–API interactions under various environmental conditions, helping identify excipients that reduce degradation pathways such as hydrolysis or oxidation [9]. Similarly, generative models are being applied to design novel polymeric or lipid-based excipients optimized for solubility enhancement and controlled release, offering opportunities beyond existing pharmacopeial excipients [60,61].

In addition, AI-driven platforms integrated with HTE and robotics accelerate the identification of optimal excipient blends. Automated laboratories can generate hundreds of formulations per day, with AI continuously updating its predictions based on real-time experimental feedback. This creates a self-learning excipient optimization system, reducing development cycles and cost [50].

Furthermore, AI facilitates the tailoring of excipient selection to patient-specific needs, an essential aspect of precision medicine. For example, predictive models can recommend lactose-free formulations for intolerant patients or suggest alternative disintegrants for pediatric versus geriatric populations [62].

### 3.3. High-Throughput Screening and Formulation Design

High-throughput screening (HTS) has been one of the essential tools in drug discovery and development, enabling researchers to test thousands of compounds and their biological activity. HTS has been revised in the formulation sciences to rapidly evaluate combinations of excipients, their stability profiles, dissolution rates, and overall manufacturability characteristics. However, traditional HTS approaches still generate vast amounts of data that require expert interpretation and can miss complex nonlinear interactions. Deep learning models now accelerate HTS by identifying promising formulation candidates with optimal performance characteristics. AI also facilitates the DoE in silico, reducing the need for exhaustive laboratory testing [2]. For example, convolutional neural networks (CNNs) and graph neural networks (GNNs) are increasingly applied to predict drug-excipient interactions directly from molecular structures, bypassing the need for exhaustive trial-and-error testing [63]. These models allow virtual pre-screening of excipient libraries, drastically reducing the required wet-lab experiments. By coupling HTS with AI, researchers can prioritize only the most promising formulations, improving both efficiency and cost-effectiveness [64].

In addition, AI enhances the DoE approaches by implementing them in silico. Traditional DoE requires predefined experimental runs to identify optimal parameter spaces. In contrast, reinforcement learning and BO approaches iteratively refine experimental design based on prior outcomes, making the process adaptive and more efficient [13]. This has been demonstrated in formulation design for certain dosage forms such as tablets and lipid-based delivery systems, where DoE guided by AI helped to reduce the experimental workload by up to 70% without comptonization in the robustness [65].

Furthermore, AI platforms coupled with robotics have assisted in establishing self-driving laboratories, where automated systems can design, execute, and analyze experiments in closed-loop workflows. In formulation science, these smart systems can optimize ratios of excipients, conditions of coating, or assembly of nanoparticles in near-real-time [66]. Notably, this approach allows bridging the gap between early-stage screening and manufacturability scalability from milligram (mg)-level prototypes to pilot-scale production.

Another key advancement with the use of AI is the use of transfer learning and multi-task DL models, which allow transfer of knowledge gained from one set of formulations (e.g., solid dispersions) to related formulation tasks (e.g., amorphous solid dispersions of poorly soluble APIs). This cross-domain learning reduces data requirements, accelerating innovation across formulation types [26]. These advancements position AI-supported HTS and DoE as foundations of next-generation pharmaceutical formulation design, combining speed, accuracy, precision, and adaptability to save time while ensuring robust, patient-centric outcomes.

### 3.4. AI in Novel Drug Delivery Systems

Liposomes are multifaceted carriers for both hydrophilic and lipophilic molecules, including small molecules, peptides, and nucleic acids [67]. However, their preparation is complex because of lipid types, physicochemical properties of the drug, and other process conditions. Conventionally, optimization of liposomal formulation requires extensive trial-and-error runs to achieve desired encapsulation, particle size, zeta potential, and in vitro or in vivo release. AI-based methods can help streamline these processes by evaluating the inputs and predicting critical formulation outcomes, thereby reducing experimental burden [52].

For example, ML algorithms such as SVMs and RF have been used to determine possible percent encapsulation efficiency based on formulation variables, including solubility, lipid-to-drug ratio, hydration method, and pH gradients [7]. These AI-based approaches help in achieving accuracies that are comparable to laboratory measurements, enabling pharmaceutical researchers to choose optimal formulation parameters prior to the scaled production. Similarly, DL models that are trained on handling high-dimensional datasets can evaluate liposome characteristics applied to predict size distributions and polydispersity indices (PDI) with high accuracy and precision [11]. This predictive capability allows better control over pharmacokinetic and, to some extent, on pharmacodynamic outcomes.

In addition, AI-assisted molecular dynamics (MD) simulations have been used as powerful tools to study lipid-to-drug interactions at the atomic level. These simulations can predict certain key answers to questions such as how hydrophobic drugs partition within the lipid bilayer, or what is the relation of cholesterol is to the rigidity of the bilayer, and how it influences the drug release profiles [68]. In recent years, AI-based MD has provided new insights into the release kinetics of anticancer agents and siRNA from PEGylated liposomes, enabling rational tuning of release rates for therapeutic efficacy.

Moreover, recent reinforcement learning and BO developments have enabled automated liposome formulation workflows. By regularly testing small batches and feeding the results back into the model, it is possible to rapidly produce formulations with desired characteristics, reducing the number of experimental repetitions by approximately 60% [69]. In conjunction with some of the advanced production methods, such as microfluidic preparation, AI-guided process optimization ensures formulation reproducibility and scalability, overcoming critical challenges in transitioning liposomal formulations from lab to clinic [70,71]. Hence, case-specific (e.g., liposomes, lipid nanoparticles, etc.) applications have demonstrated the clinical potential of AI in the formulation of novel drug delivery systems. In another study, Barenholz et al. discussed that predictive modeling has successfully optimized liposomes encapsulated with doxorubicin and paclitaxel, ensuring high percent encapsulation and minimum cardiotoxicity [72]. Similarly, in oncology and rare diseases, the AI-based design of liposomal siRNA formulations has improved stability and transfection efficiency. Overall, these advances in AI show that it is reducing complexity in novel drug formulations and accelerating clinical translation.

## 4. Dose Determination and Precision Dosing

Accurate calculation of the dose is one of the key responsibilities of pharmaceutical practice. Human or computational errors in the determination of the dose can compromise drug or formulation efficacy, cause toxicity, or reduce patient adherence to the treatment. Over the years, dosage calculations have relied on established equations, PK/PD modeling, and the experience of the clinician. With the advancement, computational systems can integrate multiple data streams, such as patient demographics, genetic factors, comorbidities, and drug–drug interactions, to optimize dosage in a patient-specific manner. Thus, AI is bridging the gap between generalized dosing guidelines and precision medicine.

### 4.1. AI Systems to Personalized Dosage Based on Patient Data

By analyzing patient data, the AI algorithms, such as ANNs and BO models, are gradually applied to individualize drug dosage. For this purpose, the system will use parameters like age, body surface area, renal function (RF) and hepatic function (HF), and genetic polymorphisms (e.g., CYP450 variants) to recommend individualized doses [73].

In cancer research, ML models have been successfully used to modify the dosing in chemotherapy to reduce the risk of toxicity and maintain dose efficacy [74]. For example, warfarin dosing has been a challenge because of its narrow therapeutic index (TI) and patient-to-patient variability. Use of AI models trained on large clinical datasets can now optimize the loading dose (LD) and maintenance doses (MDs) with higher accuracy and efficacy [75].

### 4.2. Adaptive Dosing Using Real-Time Monitoring

An adaptive dosing system that adjusts prescriptions dynamically is another example of advancement. Gadgets such as wearable sensors, continuous glucose monitors, and smart infusion pumps provide real-time PD/PK data, helping the AI algorithms to interpret these inputs to finalize dosages automatically.

As mentioned earlier, the use of AI-based wearable sensors enables continuous monitoring of therapeutic responses, enabling dose adjustments in real-time [76]. Insulin dosing for diabetics is an example that has been transformed by closed-loop systems based on AI. Moreover, “artificial pancreas” is one of the devices that maintain near-normoglycemia while reducing episodes related to hypoglycemia [77]. Alongside, some adaptive dosing models are being developed for other categories of drugs, such as antibiotics and immunosuppressants, where therapeutic drug monitoring (TDM) is critical [78,79].

### 4.3. AI in Pediatric and Geriatric Dosage Calculations

AI facilitates dosing strategies for pediatric, geriatric, and renal-impaired patients by incorporating physiological differences and metabolic profiles [80]. Dosing in special populations like children and the elderly has unique challenges. Pediatric patients require weight-based or surface area–based dosing, but some metabolic complications complicate predictions, such as ontogeny-related differences in metabolism. Geriatric patients, on the other hand, often have renal or hepatic impairment and polypharmacy concerns.

AI tools trained on pediatric pharmacokinetic datasets can now model drug clearance in neonates and infants more reliably than conventional scaling methods [78]. In geriatrics, machine learning-based decision-support systems help clinicians balance polypharmacy risks by simulating drug interactions and cumulative side effects [81].

Recent advances demonstrate that DL algorithms can integrate real-world data from EHRs, genomic profiles, and population pharmacokinetics to predict individualized dosing in these vulnerable groups. As an example, RL frameworks have been used in the optimization of antibiotic dosing in critically ill pediatric patients, which is achieved by continuously adjusting infusion rates based on patient-specific response data [82]. Similarly, AI-based geriatric pharmacology models incorporate frailty indices (FI), comorbidity patterns (CP), and certain physiologically based pharmacokinetic (PBPK) simulations to improve dose precision, accuracy, and minimize adverse drug reactions [83,84]. These types of models are expected to serve as a bridge to the translational gap between empirical guidelines and precision dosing, allowing safer and more effective pharmacotherapy across age extremes.

### 4.4. AI-Driven Dose Optimization in Clinical Trials

Clinical trials benefit significantly from AI-based dose optimization strategies. Conventionally, studies involving dose escalation follow the “3 + 3 design,” which is incompetent and may result in subtherapeutic or toxic doses. An AI-based model that uses adaptive designs, such as Bayesian Optimal Interval (BOIN) and continual reassessment methods (CRM), allows real-time adjustment of doses based on detected toxicities and efficacy signals [15].

AI-based PK/PD models use real-time patient data to determine drug concentrations and effects, adjusting doses dynamically. ANNs and BO designs are commonly used in this context [85]. Furthermore, AI-based pharmacometrics modeling fast-tracks the identification of maximum tolerated doses (MTD) and optimal biologic doses (OBD), reducing trial duration and burden on the patient [86]. Interestingly, the regulatory authorities increasingly endorse these adaptive strategies, emphasizing their growing role in designs related to next-generation clinical trials.

Overall, several AI-based dosing systems are being used in clinical decision support, extending beyond the traditional examples of warfarin and insulin. These systems are primarily applied in antibiotic therapy [87], oncology [88], anesthesia [89], and immunosuppressant dosing [90] where narrow therapeutic windows necessitate precision dosing.

### 4.5. Drug–Drug and Drug–Disease Interaction Predictions

AI systems can integrate EHRs, genomics, and literature data to assess interaction risks, ensuring safer dose regimens in polypharmacy scenarios [91]. Drug–drug interactions (DDIs) and drug–disease interactions (DDzIs) signify clinical and regulatory challenges. These interactions are responsible for a substantial part of adverse drug events, related hospitalizations, and therapeutic failures. In the past, the DDI identification methods solely relied on after-market surveillance, reports from pharmacovigilance, and other in vitro studies. However, these approaches are time-consuming, reactive, and not capable of handling the complexity of polypharmacy. AI is revolutionizing interaction prediction with its ability to mine large datasets and learn nonlinear relationships.

AI models leverage chemical structure, target binding data, gene expression, and clinical records to predict potential DDIs. Graph neural networks (GNNs) and knowledge graph–based methods integrate drug–target and protein–protein interaction data to infer novel DDIs [20]. For instance, DL models using EHR reported interactions missed by traditional pharmacovigilance, highlighting their importance in preventing adverse events [92]. Moreover, AI approaches point to the possibility of the presence of an interaction and estimate clinical outcomes such as hepatotoxicity, QT prolongation, or absence of therapeutic effect. Multi-task learning frameworks allow simultaneous prediction of multiple adverse endpoints, improving reliability [93]. In polypharmacy settings, such as oncology or geriatrics, patients may be prescribed more than 10 concurrent medications; hence multi-task learning network is particularly important.

DDzIs are equally challenging because comorbidity can alter drug safety and related efficacy. For example, β-blockers may worsen asthma, and NSAIDs may worsen peptic ulcer disease or chronic kidney disease. Use of AI algorithms that are trained on real-world evidence-based datasets can detect different patterns in how disease states affect PK/PD [94].

ML models also assist in drug repurposing by identifying unexpected beneficial DDzIs. For example, the interactions between anti-diabetic drugs and neurodegenerative disorders have been predicted by computational pipelines; this prediction is opening avenues for repositioning existing molecules [93]. In addition, AI-driven patient phenotyping enables better stratification of at-risk populations, minimizing adverse DDzIs. Modern clinical decision support systems (CDSS) incorporate AI-based DDI and DDzI predictions into platforms using electronic prescribing. These systems flag interactions, provide recommendations on dose adjustment, alternative therapy, and risk stratification tailored to individual patient comorbidities [93]. Such incorporation reduces the burden on healthcare workers, enhances patient safety, and complies with precision medicine initiatives.

## 5. AI in Quality Control and Regulatory Compliance

The pharmaceutical industry is among the most regulated sectors, with strict requirements to ensure drug quality, safety, and efficacy. Traditional quality control and regulatory workflows are labor-intensive and prone to human error, especially in pharmaceutical calculations and documentation. AI technologies are increasingly integrated into these processes to enhance efficiency, transparency, and compliance with regulatory frameworks such as FDA 21 CFR Part 11, ICH Q8–Q10, and good manufacturing practices (GMP) [95,96]. AI enables automated verification of calculations, intelligent documentation systems, and predictive quality testing. These innovations reduce human error, accelerate audits, and pave the way for real-time release testing (RTRT). AI strengthens internal quality assurance systems and regulatory interactions by combining ML, NLP, and big data analytics.

### 5.1. AI to Verify Automated Calculation

AI systems can detect and validate formulation and dose-related calculations to prevent human error, simultaneously improving consistency, regulatory, and patient compliance [97]. Accurate calculations are important for consistency in dosage, batch records, pharmaceutical manufacturing, and stability testing. Conventionally, these are performed manually or semi-manually, increasing the risk of error. AI-based systems can analyze and verify calculations in real-time; in addition, they can perform cross-checking with historical batch data [98].

For example, rule-based AI engines can ease calculations for dilution factors, excipient ratios, and potency adjustments before approval of the batch for production. ML systems also detect irregular entries in manufacturing logs, flagging potential deviations [99]. Overall, AI can minimize the loss by reducing the risks of product recalls, regulatory penalties, and adverse patient outcomes.

### 5.2. AI in Documentation and Audit Trails

NLP automates regulatory documentation, facilitating traceability and compliance with GMP [100]. A core principle in pharmaceutical compliance requires proper documentation and audit trails to ensure data integrity. AI-based systems can automate the initiation, generation, organization, and review of audit trails. This review of the audit trails will ensure compliance with ALCOA+ principles (Attributable, Legible, Contemporaneous, Original, Accurate, plus Completeness, Consistency, Enduring, and Availability) [101].

NLP tools can now automatically analyze batch production records, deviation reports, and lab notes to detect variations or missing data [101]. Moreover, predictive auditing can be achieved with AI that involves identifying trends in non-compliance before regulatory inspections. Additionally, cloud-based AI platforms can simplify secure data storage and remote audits, complying with the FDA’s digital transformation regulations [96].

### 5.3. AI Monitoring Real-Time Release Testing

By predicting critical quality attributes (CQAs) based on in-line process data, AI provides real-time release testing (RTRT), enabling faster batch releases [102]. RTRT is one of the most promising applications of AI in regulatory compliance, where traditional quality testing depends on finished product sampling, which is time-consuming and may not fully record batch-to-batch variability. AI-based RTRT systems use multivariate models incorporating sensor data, process analytical technology (PAT), and embedded spectroscopy to ensure continuous quality assurance [103].

DL algorithms can readily interpret complex spectroscopic and chromatographic data, helping manufacturers to release products based on process data rather than waiting on laboratory tests [103]. This shift supports ICH Q8–Q11 guidelines encouraging QbD approaches. Regulatory authorities, including the FDA and EMA, are highlighting the usefulness of RTRT when validated AI systems demonstrate consistent reliability [104].

The integration of RTRT accelerates product release and enhances supply chain resilience, as it minimizes batch-hold delays and reduces manufacturing costs. AI models are also being developed to predict shelf-life stability in real time, offering further regulatory and commercial benefits [105].

## 6. Challenges and Limitations in AI Usage

While AI is offering transformative potential in pharmaceutical calculations and drug development, some significant challenges still exist in its adoption. These challenges originate from limitations in data availability, the interpretability of AI models, regulatory hurdles, and infrastructure gaps within the pharmaceutical industry. Addressing these issues is critical to ensuring AI can be released safely, ethically, and effectively in healthcare and pharmaceutical sciences.

### 6.1. Data Quality and Availability

Almost all the AI models require a high-quality, diverse dataset because missing, biased, or inconsistent data can compromise model accuracy and usability [106]. AI models are only robust if the data used to train them is robust. In pharmaceutical sciences, datasets often undergo limited sample sizes, heterogeneity, and certain proprietary restrictions [106]. Furthermore, the clinical data may be incomplete, biased, or inconsistently reported across institutions, which will result in limiting the usability of AI-based predictions [107].

For example, DDI databases may lack negative examples, which can change predictive performance. Additionally, pharmaceutical research often has proprietary restrictions, which limit academic as well as cross-industry collaboration. Therefore, it is necessary to ensure use of standardized data formats, high-quality annotations, and data sharing consortia (e.g., FDA’s Sentinel Initiative) to overcome this limitation [108].

### 6.2. Interpretability and Transparency

The black-box nature of AI models, profound learning, limits interpretability, posing challenges for regulatory approval and clinical trust [109]. AI models, intense learning networks, are often criticized as “black boxes”, making it difficult to explain how predictions are generated [19]. The absence of interpretability in pharmaceutical applications, such as dosage calculation and stability prediction, weakens regulatory acceptance and clinician trust.

To provide transparency, techniques such as explainable AI (XAI), including SHAP (Shapley Additive Explanations) and LIME (Local Interpretable Model-Agnostic Explanations), are currently being explored [110]. However, one of the ongoing challenges is balancing predictive accuracy with interpretability because, without clear justifications, AI recommendations may be disregarded in clinical or regulatory decision-making [111].

### 6.3. Regulatory and Ethical Issues

In 2022, the European Medicines Agency (EMA) formally qualified Unlearn’s AI-driven approach for running smaller, more efficient clinical trials [112]. In the case of FDA, there is no well-documented case (publicly verifiable) where an AI model for formulation or dosage was fully accepted in a drug application submission as a core decision-making model [96]. The qualification in this context is a regulatory acknowledgment that a method is acceptable for use in a specific context (without full approval). It reflects a regulatory agency’s confidence in the methodology for certain applications, though it does not guarantee approval of any particular product using it. However, AI has been more commonly accepted in software-as-medical-device (SaMD) contexts (e.g., imaging, diagnostics) rather than in core drug formulation/dosage regulatory decisions [113].

It is essential to align the use of AI with the regulatory frameworks of the FDA, EMA, and ICH guidelines; however, these are still in the developing stage. In this situation, some issues like data privacy, informed consent, and liability need careful consideration [114]. Current regulatory guidelines are not fully revised to address the validation of algorithms of ML algorithms that continuously evolve with new data [115].

Additionally, some ethical issues, such as data privacy, algorithmic bias, and equitable access, need careful handling [116]. For example, some training datasets dominated by data obtained from Western populations may give biased drug-response predictions in underrepresented groups. Regulatory bodies are beginning to draft guidance documents (e.g., FDA’s 2023 AI/ML framework for drug development), but comprehensive frameworks are still under development [96].

### 6.4. Infrastructure and Skill Gaps

Implementation of AI requires skills and computational infrastructure, which may not be readily available in all pharmaceutical settings, especially in developing countries [6]. This implementation also requires significant investments in computational techniques, data storage that is secured, and integrated laboratory systems [117]. Many pharmaceutical firms, particularly small and medium enterprises (SMEs), struggle with the cost associated with the implementation of AI and associated infrastructure.

The next challenge in the implementation is the presence of a skills gap between pharmaceutical scientists and AI specialists. Bridging this gap requires an extensive training program from interdisciplinary experts, cross-functional collaborations, and reskilling of the available workforce [118]. Collaborative ecosystems involving pharmacists, clinicians, data scientists, and regulatory authorities are essential to integrating AI in pharmaceutical formulation development and dose calculations successfully [44].

Overall, it is a fact that most AI models are trained on datasets dominated by adult, Western, and homogeneous clinical data, which may contain biases in metabolic rate, genetic polymorphisms, organ maturity, and body composition that do not accurately reflect these populations. As a consequence, the dosage algorithms may under- or overestimate exposure, emphasizing the urgent need for diverse, demographically representative training data and continuous model validation across global populations [119,120]. This will include expanding training datasets to encompass data from diverse age groups, ethnicities, and geographic regions, ensuring that models capture true biological variability [121]. Additionally, implementing federated learning frameworks allows institutions worldwide to collaboratively train AI models on localized clinical data without sharing sensitive patient information, thereby improving fairness and reliability in dosage predictions [122].

## 7. Prospects and Innovations

**Figure 1:**
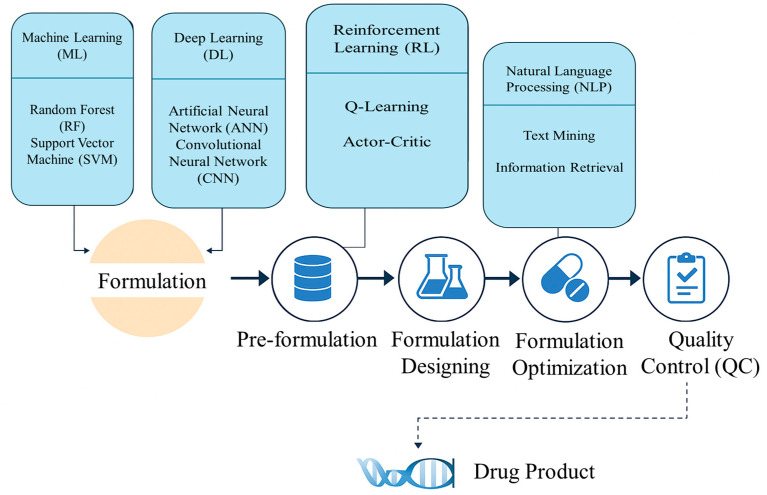
An infographic illustrating how different AI techniques (ML, DL, RL, NLP, etc.) are integrated at various stages of pharmaceutical formulation, from pre-formulation through quality control.

The future of AI in pharmaceutical calculations is in integration, personalization, and automation. The integration of AI into pharmaceutical sciences is still in its infant stage, but future directions hold positive transformative potential. Some emerging technologies (Figure 1), together with AI, can change prospects of personalized medicine, manufacturing, and clinical trial designs, redefining drug development and delivery.

### 7.1. AI with Emerging Technologies

Some of the emerging technologies include the Internet of Things (IoT), blockchain, and quantum computing. The combination of AI with these technologies will enhance data security, traceability, and computational power in the majority of pharmaceutical applications [123]. Briefly, the blockchain ensures secure as well as transparent data sharing among stakeholders; this also addresses the critical problem of data fragmentation in pharmaceutical research [124]. Meanwhile, IoT-based sensors permit real-time patient monitoring; they also provide continuous feedback for dose adjustment and safety monitoring [125]. Lastly, quantum computing is still in its experimental stage, but it shows promise for simulating molecular interactions at unprecedented speed; therefore, it may potentially revolutionize drug discovery and formulation design [126]. Additionally, coupling quantum algorithms with AI can speed up workflows in computational chemistry, particularly in drug–receptor binding. These advances highlight how AI will not act alone but, instead, will be integrated with other emerging technologies to transform pharmaceutical sciences.

### 7.2. 3D-Printed and Personalized Pharmaceuticals

AI-driven models optimize formulations for 3D printing technologies, enabling on-demand and patient-specific drug products [127]. The rise of 3D printing (additive manufacturing) offers opportunities to fabricate patient-specific dosage forms, with AI algorithms guiding dose optimization, excipient selection, and geometry design [128]. For instance, the prediction of disintegration time, mechanical strength, and release kinetics of 3D-printed tablets can be achieved using AI-based design models, which will tailor drug delivery profiles [129].

Personalized pharmaceuticals will benefit from AI’s ability by integrating genomic, phenotypic, and clinical data. This integration will allow the production of precision-matched therapeutics, where patients would receive formulations designed for their unique metabolism and other therapeutic needs [130]. Regulatory agencies are beginning to explore frameworks for 3D-printed pharmaceuticals, suggesting that this innovation may soon become mainstream [131].

### 7.3. AI in Clinical Trials and Drug Repurposing

Based on multi-omics and real-world data, AI can accelerate the identification of novel candidates and predict clinical outcomes [132]. Hence, AI holds promising potential in drug repurposing and redesigning clinical trials, making them faster, cheaper, and more adaptive. As mentioned earlier in this review, the traditional randomized controlled trials are time-consuming and not cost-effective; AI permits virtual trial simulations and adaptive designs that respond to interim data [133]. For example, ML models can perform real-time monitoring of safety signals, predict the risk of patient dropout, and can be used for optimization of recruitment.

Drug repurposing is another area where AI is promising because it can mine the biomedical literature, related databases, and EHR, plus it can identify new therapeutic uses for existing drugs. This accelerates innovations in pharmaceutical sciences, simultaneously reducing development costs and risks. Several AI-based repurposing efforts during the COVID-19 pandemic underscored the potential of this approach [134]. Therefore, some emerging platforms such as Benevolent AI and in silico Medicine show the capability to propose novel repurposing candidates [135].

### 7.4. Predictive Toxicology and Safety Assessment

Artificial intelligence is transforming preclinical toxicology by predicting potential adverse effects of new chemical entities before in vivo testing. ML and DL models trained on large toxicogenomic and cheminformatics datasets can identify structural alerts and forecast organ-specific toxicity, genotoxicity, and hepatotoxic potential [136]. For example, graph neural networks and transformer-based architectures have demonstrated superior accuracy in predicting drug-induced liver injury (DILI) compared to conventional QSAR models [137]. The integration of AI-based predictive toxicology into formulation pipelines accelerates early-stage screening and reduces animal use, costs, and attrition rates during the drug development process.

### 7.5. Quantum Computing and AI-Enhanced Molecular Simulation

The convergence of AI and quantum computing presents a new frontier for simulating molecular interactions at unprecedented precision. Quantum machine learning (QML) algorithms can process complex molecular orbitals and energy states, enabling more accurate prediction of drug and target binding affinities [138]. When integrated with AI-based molecular dynamics simulations, these approaches offer the potential to design optimized formulations and delivery systems at the atomic scale [139]. As quantum processors evolve, their synergy with AI can revolutionize computational, biopharmaceutical, and clinical pharmaceutics by reducing the time and cost associated with hit-to-lead optimization and formulation design, useful for special populations.

## 8. Conclusions

AI is entering as a transformative force in pharmaceutical sciences, particularly drug formulation and dosage calculations. By leveraging ML, DL, and predictive modeling, AI helps researchers and clinicians to process vast datasets, extract meaningful insights, and based on this, they can design optimized formulations more accurately and efficiently. Some applications, such as predicting drug-to-excipient compatibility, optimization of dose, quality control, and drug-to-drug interactions, demonstrate that AI is not a futuristic concept but a practical tool that can reshape the pharmaceutical landscape. Despite these advances, some significant challenges still exist, such as issues related to data quality, algorithm interpretability, regulatory compliance, and ethical use. As mentioned earlier in this review, the AI models can only be robust if they are trained robustly. Incomplete or biased datasets can lead to incomplete or inaccurate predictions, leading to potentially serious clinical implications. Moreover, regulatory frameworks for the use of AI in drug development and clinical practice have not yet been fully revised. The establishment of such frameworks will require interdisciplinary collaboration between technologists, pharmaceutical scientists, and policymakers.

Integration of AI with emerging technologies, such as blockchain, IoT, quantum computing, and 3D printing, holds enormous promise. This coupling will be useful particularly in areas like personalized pharmaceuticals, adaptive clinical trials, and precision dosing strategies tailored to individual patient needs. AI-based drug repurposing and real-time monitoring of treatment outcomes will ease the transition toward precision medicine, bringing cost reductions while improving patient outcomes. Ultimately, the success of AI in pharmaceutical sciences will depend on nurturing interdisciplinary collaboration. Therefore, computational scientists, pharmacists, clinicians, and regulatory experts must collaborate to achieve the safe, ethical, and effective deployment of AI-based solutions. If we overcome these challenges, AI has the potential to revolutionize the pharmaceutical industry, piloting a new era of patient-centered, efficient, and innovative healthcare.

## Figures and Tables

**Table 1:** Summarizes key AI approaches, their applications, and supporting references.

AI Technique	Use in Drug Formulation/Dosage Calculations	Reference
Artificial Neural Networks (ANNs)	Prediction of solubility, dissolution rates, encapsulation efficiency, drug-to-excipient ratios, and estimation of pharmacokinetic parameters.	[2,3]
Support Vector Machines (SVMs)	Classify excipients by compatibility, predict encapsulation efficiency in novel drug delivery systems such as liposomes and nanoparticle systems.	[7,8]
Random Forest (RF)	Selection for formulation parameters; prediction of optimal excipient concentrations for stability and bioavailability.	[9,10]
Deep Learning (DL, CNN/GNN)	Prediction of particle size, PDI, and drug release;, modeling complex nonlinear relationships in formulation datasets.	[11,12]
Bayesian Optimization	Optimization of dose, excipient concentration refinement, and efficient design of experiments (DoE).	[13,14]
Reinforcement Learning (RL)	Dosing strategies (e.g., insulin pumps), optimizing trial design for dose-escalation studies.	[15,16]
Natural Language Processing (NLP)	Extracts dosage guidelines, stability data, and drug interactions from literature.	[3,17,18]
Explainable AI (XAI)	Improves interpretability of dose predictions; regulatory acceptance.	[1,19]
Knowledge Graphs (KGs)	Predicts DDIs and DDzIs; supports polypharmacy dosage adjustments.	[20,21]
Graph Neural Networks (GNNs)	Drug–excipient compatibility; formulation stability prediction.	[22,23]
Generative Adversarial Networks (GANs)	Generate novel molecules; simulate formulation outcomes.	[24,25]
One-Shot/Few-Shot Learning	Dose–response prediction from limited data.	[26,27]
Transfer Learning	Improves model performance with small pharmaceutical datasets.	[28,29,30]
Federated Learning	Enables multi-institutional modeling without data sharing.	[31,32]
Hybrid ML–QbD Models	Combines AI with QbD for robust formulations.	[1,33,34]
Deep Reinforcement Learning	Optimizes trial designs and adaptive dosing.	[1,35,36]
Multimodal Learning	Combines chemical, imaging, omics, and text data for prediction.	[37,38]
AutoML (Automated ML)	Selects the best algorithms/features for dosage models automatically.	[39,40]
Digital Twins	Patient-specific simulation of drug response for precision dosing.	[41,42]

## Abbreviations

The following abbreviations are used in this manuscript:

AI	Artificial Intelligence
ANN	Artificial Neural Network
API	Active Pharmaceutical Ingredient
BO	Bayesian Optimal Interval
CDSS	Clinical Decision Support System
CNN	Convolutional Neural Network
CQAs	Critical Quality Attributes
CRM	Continual Reassessment Method
DDIs	Drug–Drug Interactions
DDzIs	Drug–Disease Interactions
DL	Deep Learning
DoE	Design of Experiment
EHRs	Electronic Health Records
GMP	Good Manufacturing Practices
GNN	Graph Neural Network
HTE	High-Throughput Experimentation
HTS	High-Throughput Screening
IoT	Internet of Things
LIMS	Laboratory Information Management System
ML	Machine Learning
MTD	Maximum Tolerated Dose
NLP	Natural Language Processing
OBD	Optimal Biologic Dose
PAT	Process Analytical Technology
PD	Pharmacodynamics
PDI	Polydispersity Index
PK	Pharmacokinetics
QSAR	Quantitative Structure–Activity Relationship
QbD	Quality by Design
RTRT	Real-Time Release Testing
SVM	Support Vector Machine
TDM	Therapeutic Drug Monitoring
XAI	Explainable Artificial Intelligence
